# Neuropeptide Y Overexpressing Female and Male Mice Show Divergent Metabolic but Not Gut Microbial Responses to Prenatal Metformin Exposure

**DOI:** 10.1371/journal.pone.0163805

**Published:** 2016-09-28

**Authors:** Henriikka Salomäki-Myftari, Laura H. Vähätalo, Liisa Ailanen, Sami Pietilä, Asta Laiho, Arno Hänninen, Juha-Pekka Pursiheimo, Eveliina Munukka, Anniina Rintala, Eriika Savontaus, Ullamari Pesonen, Markku Koulu

**Affiliations:** 1 Institute of Biomedicine, Department of Pharmacology, Drug Development and Therapeutics, University of Turku, Turku, Finland; 2 Drug Research Doctoral Programme (DRDP), University of Turku, Turku, Finland; 3 Bioinformatics Unit, Turku Centre for Biotechnology, University of Turku and Åbo Akademi University, Turku, Finland; 4 Institute of Biomedicine, Department of Medical Microbiology and Immunology, University of Turku, Turku, Finland; 5 Turku Clinical Sequencing Laboratory, Institute of Biomedicine, University of Turku, Turku, Finland; Institut d'Investigacions Biomèdiques August Pi i Sunyer, SPAIN

## Abstract

**Background:**

Prenatal metformin exposure has been shown to improve the metabolic outcome in the offspring of high fat diet fed dams. However, if this is evident also in a genetic model of obesity and whether gut microbiota has a role, is not known.

**Methods:**

The metabolic effects of prenatal metformin exposure were investigated in a genetic model of obesity, mice overexpressing neuropeptide Y in the sympathetic nervous system and in brain noradrenergic neurons (OE-NPY^DβH^). Metformin was given for 18 days to the mated female mice. Body weight, body composition, glucose tolerance and serum parameters of the offspring were investigated on regular diet from weaning and sequentially on western diet (at the age of 5–7 months). Gut microbiota composition was analysed by 16S rRNA sequencing at 10–11 weeks.

**Results:**

In the male offspring, metformin exposure inhibited weight gain. Moreover, weight of white fat depots and serum insulin and lipids tended to be lower at 7 months. In contrast, in the female offspring, metformin exposure impaired glucose tolerance at 3 months, and subsequently increased body weight gain, fat mass and serum cholesterol. In the gut microbiota, a decline in *Erysipelotrichaceae* and *Odoribacter* was detected in the metformin exposed offspring. Furthermore, the abundance of *Sutterella* tended to be decreased and *Parabacteroides* increased. Gut microbiota composition of the metformin exposed male offspring correlated to their metabolic phenotype.

**Conclusion:**

Prenatal metformin exposure caused divergent metabolic phenotypes in the female and male offspring. Nevertheless, gut microbiota of metformin exposed offspring was similarly modified in both genders.

## Introduction

Metformin, a biguanide, is the first-in-line medication for type 2 diabetes [1,2]. Metformin alleviates hyperglycemia essentially by lowering hepatic glucose production [3]. Additionally, metformin has a variety of pleiotropic effects including improved lipid and cholesterol metabolism, decreased inflammation and inhibition of cell growth [4]. Nowadays, metformin is receiving increased acceptance as a treatment for gestational diabetes mellitus (GDM) by minimizing the adverse effects of maternal hyperglycemia to the fetus [5,6]. A recent clinical meta-analysis of pharmacological treatment regimen in GDM concluded metformin to have a favorable profile on maternal and early infant parameters [7]. Moreover, the use of metformin on obese, non-diabetic mothers has also been evaluated in a topical clinical trial [8]. On the other hand, the use of metformin during pregnancy has raised concerns of its long-term effects. Few studies have now shown increased body weight of metformin exposed infants [9,10] and one has reported higher fasting glucose and a tendency to elevated blood pressure in young metformin exposed children [11]. All in all, the data on the long-term effects of the exposure in humans is still scarce and only few animal studies on these effects have been performed [12–14]. Our own results have shown that prenatal metformin exposure prevents obesity-prone phenotype in a model where the dams were fed with high fat diet [14]. Moreover, the gene expression of the mitochondrial respiration pathway was altered in the liver and adipose tissue of the neonatal pups linking the early transcriptome to the later phenotype. However, we have also shown that prenatal metformin exposure in metabolically normal dams leads to diet-induced obesity, the mechanism possibly owing to an induction of starvation-like situation during gestation [12,15].

Gut microbiota has received a lot of attention for its connection to metabolic processes on the whole body level [16]. Intriguingly, gastrointestinal track and gut microbiota have also been proposed to play a role in the pharmacological actions of metformin [17–20]. Metformin treatment has been shown to alter the metabolism of *E*.*coli* in *C*.*elegans* [21], increase the level of metabolically beneficial *Akkermansia muciniphila* bacterial species in mice [22,23] and short chain fatty acid (SCFA) producing microbiota in rats [24]. Moreover, studies with type 2 diabetics have shown that metformin treatment associates with gut microbial changes [19] and that metformin causes a distinct footprint on the gut microbiota that can be connected to the adverse as well as to the therapeutic effects of the drug [25]. In light of growing evidence of the connection of metformin to gut microbiota, we examined whether the long-term effects of prenatal metformin are conveyed through changes in the gut microbiota of the offspring. Modulations of the offspring’s microbiota with accompanying changes in the inflammatory responses and metabolism have previously been introduced by antibiotic treatment [26] and western-type diet [27] during gestation.

In this study, we used homozygous transgenic mice overexpressing neuropeptide Y (NPY) in the peripheral sympathetic nerves and in the brain noradrenergic neurons under the dopamine beta-hydroxylase promoter (OE-NPY^DβH^ mouse) [28,29]. NPY is a 36-amino acid neurotransmitter expressed abundantly in the brain and in the sympathetic nervous system and acts as a key regulator of energy balance [30,31]. Excessive central NPY leads to metabolic effects such as increased weight gain, adiposity and hyperinsulinemia [32,33]. Moreover, peripheral NPY has a fundamental role in stress-induced obesity, i.e. NPY is needed to convey the effects of stress on fat storage, adipogenesis and corticosterone secretion [34,35]. In humans, a gain-of-function mutation of Leucine 7 to Proline 7 (p.L7P) in the signal peptide of the preproNPY leads to increased release of NPY upon sympathetic stimulation [36] and has been shown to associate with several metabolic traits such as higher cholesterol [37], accelerated atherosclerosis [38] increased BMI [39], risk for type 2 diabetes [40] and diabetic retinopathy [41,42]. The OE-NPY^DβH^ mouse model reflects the phenotypic effects of p.L7P polymorphism in humans [28,29]. Both heterozygous and homozygous OE-NPY^DβH^ mice become obese and develop impairments in the glucose homeostasis with age on a normal chow diet [28,29]. The metabolic impairments of OE-NPY^DβH^ are attributed to increased fat storage, changes in the sympathetic tone and brown adipose tissue thermogenesis without increased food intake or decreased energy expenditure [28,29].

Therein, we hypothesized that prenatal metformin exposure protects the offspring from obesity and metabolic disturbances also in the OE-NPY^DβH^ mouse. Furthermore, we proposed that NPY overexpression affects the composition of the gut microbiota which may further be modulated by prenatal metformin exposure. As the gut microbiota of the OE-NPY^DβH^ mouse has not been investigated earlier, a genotype comparison between OE-NPY^DβH^ and WT mice was included in our study. By investigating the gut microbiota at the age when the metabolic phenotype of the OE-NPY^DβH^ mouse outbursts [29], we aimed to identify microbial alterations that are induced by metformin exposure and are not confounded by impaired metabolism.

## Methods

### Animals

Homozygous transgenic OE-NPY^DβH^ mice overexpressing NPY in the peripheral sympathetic nerves and in the brain noradrenergic neurons [28,29] and C57BL/6N wild-type (WT) mice originating from the same heterozygous litters were used. The mice were housed on a 12 h:12 h dark:light cycle and food and water were available *ad libitum* unless otherwise stated. Animal work was planned and performed according to the Act on the Protection of Animals Used for Scientific or Educational Purposes. The study scheme was approved by the Finnish Animal Experiment Board (Permit ESAVI-2010-06188). The mice were monitored for any signs of morbidity and all efforts were made to minimise suffering.

### Study design

Homozygous OE-NPY^DβH^ female mice (10–12 weeks) were let to mate three consecutive days with homozygous OE-NPY^DβH^ male mice. Age-matched WT mice and their offspring were included in the study to verify the metabolic OE-NPY^DβH^ phenotype as it has been previously reported [29] and to reveal novel findings on the effects of NPY overexpression on gut microbiota. Metformin (300 mg/kg, Sigma-Aldrich, St. Louis, MO, USA) or vehicle for the OE-NPY^DβH^ mice and vehicle for the WT mice was given by oral gavage in the morning after the first mating day and continued for the following 18 days. The dose of metformin was selected according to previous studies [12,13,18,43–45]. Body weight and food intake was measured daily during the administration. The vehicle (VEH) and metformin (MET) exposed OE-NPY^DβH^ offspring and VEH exposed WT offspring were fed with regular chow diet (RD; CRM(E), SDS, UK) until 5 months of age and thereafter a western diet (WD: 21% fat, 0.15% cholesterol and 19.5% casein (% as w/w); Altromin, Germany) until 7 months of age. In all experiments, results on both male and female offspring are reported.

### Glucose metabolism

Fasting blood glucose during the gestation was measured (Precision Xceed, Abbot Diabetes Care Ltd, Oxon, UK) on the 11-13^th^ day of administration. Pregnant mice were administrated with MET or VEH and fasted for 4 hours (8.00 a.m-12.00 p.m.) prior to glucose measurement. Offspring’s glucose tolerance was tested at 3 and 6 months during RD and WD, respectively. Mice were fasted for 4 hours and blood glucose was measured prior to and 20, 40, 60 and 90 minutes after i.p. glucose administration (1 g/kg). Additionally, blood glucose of the offspring was measured from the tail vein after 3 hours fasting at 7 months of age. Insulin was measured (Ultrasensitive mouse insulin, Mercodia, Uppsala, Sweden) from the serum of anesthetized mice after 3 hours fasting at 7 months of age. Fasting glucose and insulin values were further used for homeostatic model assessment of insulin resistance (HOMA-IR: glucose mmol/l x insulin μU/ml/22.5), beta-cell function (HOMA-β: 20 x insulin μU/ml / (glucose mmol/l– 3.5)) and quantitative insulin sensitivity check index (QUICKI; 1 / (log(insulin μU/ml) + log(glucose mg/dl)).

### Body composition

Body composition (i.e. fat and lean mass) was measured by quantitative nuclear magnetic resonance (NMR) scanning (EchoMRI-700, Echo Medical Systems) at 4 and 7 months during RD and WD, respectively.

### Tissue collection

Terminal anesthesia was induced as previously described [12] and blood was obtained *via* cardiac puncture. Subsequent to decapitation, the following tissues were weighed and collected: liver, brown adipose tissue (BAT), inguinal (iWAT), gonadal and epididymal (gWAT and eWAT, respectively), mesenteric (mWAT) and retroperitoneal white adipose tissue (rWAT).

### Serum lipids

Serum triglycerides, non-esterified fatty acids (NEFA) and total cholesterol were quantified as previously described [12].

### Gut microbiota analyses

Voluntarily voided fecal samples were collected from 10-11-week-old VEH and MET exposed OE-NPY^DβH^ and VEH exposed WT offspring during RD. The samples were taken from 3–5 cages/group to avoid sample homogeneity resulting from the same cage environment and the samples were stored at -70°C. DNA was extracted from the weighed, homogenized fecal pellets with semi-automated GXT stool extraction kit (Hain Lifescience, Nehren, Germany). Prior to the extraction, mechanical lysis was performed by bead-beating the samples in glass bead tubes with MOBIO PowerLyzer^™^ 24 Bench Top Bead-Based Homogenizer. The DNA concentrations were measured with Qubit 2.0 dsDNA HS assay kit (Life Technologies), after which the DNAs were stored at -80°C.

The sequencing libraries for NGS-based gut microbiota composition analysis were generated in a single PCR with custom dual-indexed primers containing the adapter and specific index sequences required for sequencing. Briefly, the V4-V5 area of the bacterial 16S rRNA gene was amplified using KAPA HiFi PCR kit (KAPA Biosystems) with in-house generated primers modified from [46]. The forward and reverse primer sequences were 5’AATGATACGGCGACCACCGGATCTACi5TATGGTAATTGTGTGCCAGCMGCCGCGGTAA-3’ and 5’-CAAGCAGAAGACGGCATACGAGATi7AGTCAGTCAGGCCCCGTCAATTCMTTTRAGT-3’, respectively, where i5 and i7 represent the 8-nucleotide index sequences enabling the identification of the sequences originating from each original sample. The PCR products were purified with Agencourt AMPure XP Magnetic beads (Beckman Coulter, Inc.) on DynaMag^™^-96 magnetic plate (Life Technologies). The PCR product length and DNA integrity were checked with TapeStation (Agilent Technologies Inc.), and the final DNA concentrations of the purified products were measured with Qubit 2.0 dsDNA HS assay kit (Life Technologies). The products were then mixed in equal concentrations to generate a 4 nM library pool, which was denatured [47], diluted into a final concentration of 4 pM, and spiked with 25% denatured PhiX control (Illumina) for sequencing.

Raw reads across the 35 samples sequenced with the Illumina MiSeq 300bp paired-end sequencing were used as input for the data analysis. The quality of the raw reads was checked with FastQC (v.0.10.1) after which the downstream analysis was carried out using Qiime (v.1.9.1). Reads were first quality filtered requiring at least 20 Phred quality score, resulting in 60k–978k reads per sample (mean: 127k, sd: 188k). Chimeric sequences were filtered using usearch (v.6.1). The chimera sequence check was carried out against GreenGenes database (v. 13.08). Operational Taxonomic Units (OTUs) were picked using uclust clustering method with 97% sequence similarity and OTUs with less than 0.05% of total sequence count were removed. Annotations for the resulting OTUs were derived from the GreenGenes database. Qiime was also used for the Principal Coordinate analysis (PCoA) based on the beta diversity measures of the OTUs. Taxonomic summary produced by Qiime was visualized as bar charts on the phylum level. The samples were subsampled (rarefied) by random sampling without replacement to the lowest common sequencing depth. Functional profiling of the microbiota data was performed with PICRUSt (phylogenetic investigation of communities by reconstruction of unobserved states), a method and its rationale described by Langille et al. [48]. The workflow can be found also from https://picrust.github.io/picrust/tutorials/metagenome_prediction.html#metagenome-prediction-tutorial. The predicted pathways were analysed by Mann-Whitney U-test combined with false discovery rate (FDR; Benjamini-Hochberg procedure) using the program R (http://www.r-project.org/).

### Statistical analyses

The statistical analyses were performed with GraphPad Prism 6.0 and program R. Glucose tolerance and body weight development were analysed with two-way repeated measures-ANOVA (2-way RM-ANOVA) with Sidak’s or Tukey’s post-hoc tests. In the body weight development data, 6 values (4 in male offspring and 2 in female offspring) at week 13 were mathematically predicted with linear regression model in the program R in order to obtain the full data for statistical analyses.

The effect of prenatal metformin exposure on the OE-NPY^DβH^ mice (VEH OE-NPY^DβH^ vs. MET OE-NPY^DβH^) and the effect of the transgene (OE-NPY^DβH^ vs. WT) were tested separately with Student’s t-test or Mann-Whitney test depending on the normality distribution of the data. 2-way ANOVA was used for microbiota analyses when assessing the effect of prenatal metformin exposure in both genders. Pearson and Spearman correlations were used for assessing correlation. Results were considered statistically significant if P < 0.05.

## Results

### Metabolic status and metformin treatment of the OE-NPY^DβH^ dams

To determine the metabolic status of the OE-NPY^DβH^ mice, WT mice were used as an internal control. Body weight of the 10–12 week-old OE-NPY^DβH^ and WT female mice designated for mating was equal and the mice were normoglycemic by fasting glucose level in both groups (WT: 7.4 ± 0.3 mmol/l; OE-NPY^DβH^: 7.6 ± 0.3 mmol/l). The weight of the vehicle treated OE-NPY^DβH^ and WT dams also developed similarly during the gestation (data not shown). Glucose tolerance was not tested during the gestation to avoid excess stress. However, a separate study with non-pregnant mice showed that OE-NPY^DβH^ female mice have a tendency to impaired glucose tolerance (P = 0.06) at 11 weeks of age and a tendency to increased fat mass% (WT 18.9 ± 1.0%, OE-NPY^DβH^ 21.6 ± 1.0%; P = 0.07) at 16 weeks of age suggesting that the time of the gestation collides with the window of onset for the metabolic phenotype.

**Fig 1:**
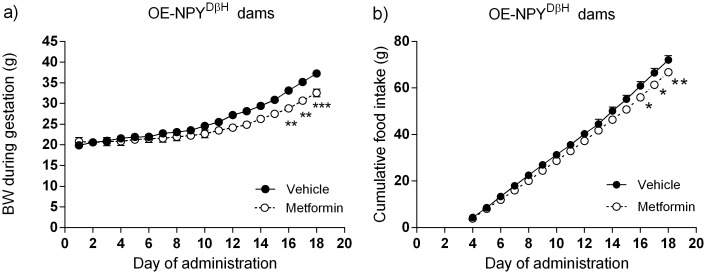
Effects of metformin treatment on the OE-NPY^DβH^ dams. Body weight (BW, a) and cumulative food intake (b) of the metformin and vehicle treated OE-NPY^DβH^ dams measured during the gestation on the day of metformin or vehicle administration. n = 4–5, significances by 2-RM-ANOVA with Sidak’s multiple comparisons test (a, b). The data expressed as mean ± SEM, *P < 0.05, **P < 0.01 and ***P < 0.001.

Metformin treatment in the OE-NPY^DβH^ dams reduced their body weight gain with statistical significance during the last three days of administration in comparison to the vehicle treatment (Fig 1a). Furthermore, metformin treated OE-NPY^DβH^ dams showed also decreased food intake (Fig 1b). There was no significant difference in the litter sizes. The fasting glucose rose slightly in the metformin treated OE-NPY^DβH^ dams during the gestation but this effect was most probably not a direct effect of metformin due to a lower baseline blood glucose in the OE-NPY^DβH^ female mice designated to metformin (baseline of 7.6 ± 0.3 mmol/l and mid-gestational value of 8.2 ± 0.3 mmol/l in the vehicle treated OE-NPY^DβH^ dams; 6.9 ± 0.4 and 8.2 ± 0.3 mmol/l in the metformin treated OE-NPY^DβH^ dams, respectively).

### Body weight and body composition of the offspring

**Fig 2:**
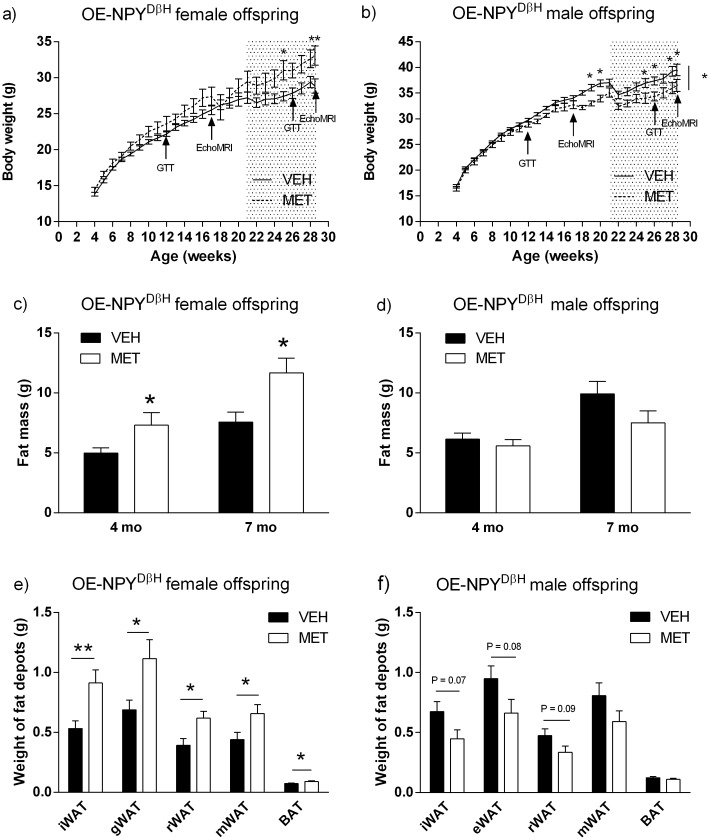
Body weight development and body composition of the VEH and MET exposed OE-NPY^DβH^ offspring. Body weight development of the female (a) and male (b) offspring. GTT and EchoMRI marked on the figures. The gray shaded area = western diet (WD). Fat mass (FM; g) of the VEH and MET exposed OE-NPY^DβH^ female (c) and male (d) offspring at 4 (during RD) and 7 months (during WD). The weight of inguinal^#^ (iWAT), gonadal/epididymal (gWAT/eWAT), retroperitoneal (rWAT), mesenteric (mWAT) white adipose tissue and brown adipose tissue (BAT) of the female (e) and male (f) offspring at 7 months. n(females) = 11 in VEH and 8 in MET exposed offspring and n(males) = 20 in VEH and 14 in MET exposed offspring. ^#^n = 13 in iWAT of the MET exposed male offspring. Significances by 2-RM-ANOVA and Sidak’s multiple comparisons test (a, b) and Student’s t-test (c-f). The data expressed as mean ± SEM, *P < 0.05, **P < 0.01 and ***P < 0.001.

The body weight and body composition data of the VEH exposed OE-NPY^DβH^ and WT offspring are presented in Table 1 and show the expected genotype effect in the OE-NPY^DβH^ mice with impaired glucose tolerance and increased fat mass. MET exposure increased body weight gain of the OE-NPY^DβH^ female offspring in comparison to VEH exposed OE-NPY^DβH^ female offspring and significant differences occurred during the WD period (2-way RM-ANOVA, Fig 2a). On the contrary, MET exposed OE-NPY^DβH^ male offspring showed decreased weight gain compared to the VEH exposed OE-NPY^DβH^ male offspring (Fig 2b). Fat mass (g) of the MET exposed female offspring was increased at 4 and 7 months (Fig 2c) while there was no difference in the lean mass (18.5 ± 0.3 g and 18.3 ± 0.3 g for VEH and MET exposed female offspring at 4 months and 19.1 ± 0.2 g and 19.3 ± 0.3 g for VEH and MET exposed female offspring at 7 months, respectively). Thus, the excess weight was due to increased adiposity. Accordingly, WAT depots (iWAT, gWAT, rWAT and mWAT) and BAT were heavier at 7 months (Fig 2e). There was also a tendency towards increased liver weight (1.09 ± 0.02 g and 1.18 ± 0.04 for VEH and MET exposed female offspring respectively; P = 0.065). In the MET exposed male offspring, fat mass was observably declined at 7 months but this did not reach significance (P = 0.12, Fig 2d). At 4 months, lean mass was 24.3 ± 0.2 g and 23.8 ± 0.3 g and at 7 months 26.0 ± 0.2 g and 25.7 ± 0.3 g for VEH and MET exposed male offspring, respectively. There was a tendency to a lower weight in iWAT (P = 0.071), eWAT (P = 0.078) and rWAT (P = 0.094) in the MET exposed male offspring. However, BAT (Fig 2f) nor the liver weight was changed (liver: 1.51 ± 0.06 g and 1.51 ± 0.04 g for VEH and MET exposed male offspring, respectively).

**Table 1:** Metabolic parameters of the VEH exposed OE-NPY^DβH^ and WT female and male offspring at 3, 4, 6 and 7 months.

	Female offspring			Male offspring		
	WT (*n*)	OE-NPY^DβH^ (*n*)	P-value	WT (*n*)	OE-NPY^DβH^ (*n*)	P-value
**3 months:**						
**GTT (AUC)**	836 ± 13 (17)	890 ± 26 (11)	< 0.05	839 ± 28 (15)	949 ± 33 (20)	< 0.01
**4 months:**						
**Body weight (g)**	24.7 ± 0.2 (17)	25.5 ± 0.7 (11)	0.158	31.3 ± 0.3 (16)	34.0 ± 0.5 (20)	< 0.001
**FM (g)**	3.0 ± 0.1 (17)	5.0 ± 0.4 (11)	< 0.001	3.6 ± 0.4 (16)	6.1 ± 0.5 (20)	< 0.001
**LM (g)**	19.7 ± 0.2 (17)	18.5 ± 0.3 (11)	< 0.001	24.5± 0.3 (16)	24.3 ± 0.2 (20)	0.581
**6 months:**						
**GTT (AUC)**	859 ± 17 (17)	1070 ± 43 (11)	< 0.001	899 ± 70 (15)	1059 ± 43 (20)	0.05
**7 months:**						
**Body weight (g)**	26.9 ± 0.3 (17)	28.9 ± 1.0 (11)	< 0.05	34.3 ± 0.7 (16)	39.5 ± 1.1 (20)	< 0.001
**FM (g)**	4.0 ± 0.3 (17)	7.6 ± 0.8 (11)	< 0.001	5.7 ± 0.9 (16)	9.9 ± 1.0 (20)	< 0.01
**LM (g)**	20.7 ± 0.1 (17)	19.1 ± 0.2 (11)	< 0.001	25.5 ± 0.3 (16)	26.0 ± 0.2 (20)	0.216
**Triglycerides (mg/ml)**	0.28 ± 0.02 (17)	0.32 ± 0.03 (11)	0.164	0.35 ± 0.03 (16)	0.34 ± 0.02 (20)	0.604
**NEFA (mmol/l)**	0.36 ± 0.04 (15)	0.35 ± 0.03 (11)	0.960	0.37 ± 0.04 (8)	0.41 ± 0.03 (20)	0.462
**Cholesterol (mmol/l)**	1.53 ± 0.06 (17)	1.60 ± 0.08 (11)	0.534	2.41 ± 0.11 (16)	2.64 ± 0.21 (20)	0.362
**Glucose (mmol/l)**	7.3 ± 0.3 (17)	7.8 ± 0.4 (11)	0.434	7.3 ± 0.3 (16)	8.3 ± 0.5 (20)	0.151
**Insulin (μg/l)**	0.16 ± 0.04 (8)	0.21 ± 0.03 (11)	0.371	0.22 ± 0.05 (8)	0.46 ± 0.06 (17)	< 0.05

GTT = glucose tolerance test, AUC = area under the curve, FM = fat mass, LM = lean mass, NEFA = non-esterified fatty acids. Values expressed as mean ± SEM. Number of mice in each experiment indicated in the parenthesis. P-values by Student’s t-test, Mann-Whitney U-test or 2-way RM-ANOVA (GTT).

### Glucose homeostasis

**Fig 3:**
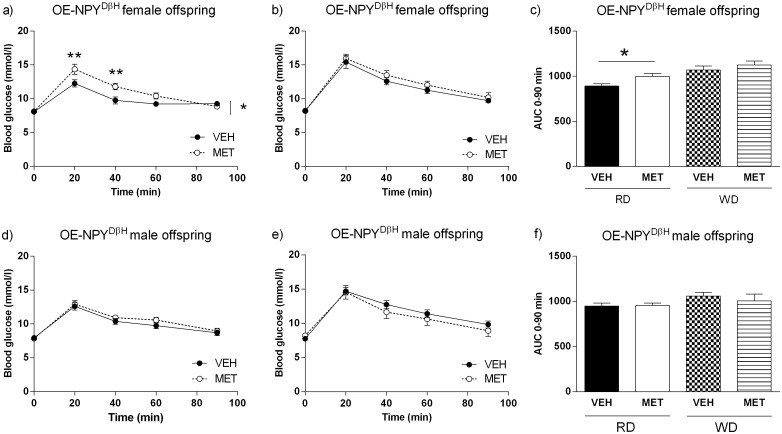
Glucose homeostasis. Glucose tolerance test of the VEH and MET exposed OE-NPY^DβH^ female and male offspring at 3 months during RD (a, d) and at 6 months during WD (b, e), respectively. Corresponding AUC values of the GTTs (c, f). n(females) = 11 in VEH and 8 in MET exposed offspring and n(males) = 20 in VEH and 13 in MET exposed offspring. Significances by 2-RM-ANOVA (a, b, d, e) and Student’s t-test (c, f). The data expressed as mean ± SEM, *P < 0.05, **P < 0.0.1.

NPY overexpression caused impaired glucose tolerance during RD and WD in both genders (Table 1). MET exposure in the OE-NPY^DβH^ female offspring worsened glucose tolerance during RD exceeding the effect of NPY alone (Fig 3a and 3c). Glucose tolerance did not correlate with body weight (Pearson P = 0.79 and 0.54 for VEH and MET exposed female offspring, respectively) or fat mass at 4 months (Pearson P = 0.70 and 0.63 for VEH and MET exposed female offspring, respectively). WD abrogated the difference and at 6 months, glucose tolerance was similar between VEH and MET exposed OE-NPY^DβH^ female offspring (Fig 3b and 3c). However, at this time point, glucose tolerance correlated with fat mass (7 months) only in the VEH exposed OE-NPY^DβH^ offspring (Pearson P < 0.05 and 0.61 for VEH and MET exposed offspring, respectively).

Metformin exposure did not significantly affect glucose tolerance of the OE-NPY^DβH^ male offspring either during RD or WD (Fig 3d–3f). Nevertheless, fasting insulin at 7 months tended to be lower in the MET exposed male offspring (P = 0.08, Table 2) and HOMA-β index calculated based on the fasting glucose and insulin level implicated that prenatal metformin exposure alleviated insulin resistance in the OE-NPY^DβH^ male offspring during WD (HOMA-β index P < 0.05, Table 2). Furthermore, glucose tolerance at 6 months correlated with fat mass (7 months) (Pearson P < 0.001 for VEH and MET exposed male offspring). Consequently, insulin levels at 7 months correlated with corresponding fat mass (Pearson P < 0.01 and < 0.001 for VEH and MET exposed male offspring, respectively; data not shown).

**Table 2:** Comparison of the serum profile, HOMA-IR, HOMA-β and QUICKI of the VEH and MET exposed OE-NPY^DβH^ offspring at 7 months.

	OE-NPY^DβH^ female offspring			OE-NPY^DβH^ male offspring		
	VEH (*n*)	MET (*n*)	P-value	VEH (*n*)	MET (*n*)	P-value
**Triglycerides (mg/ml)**	0.32 ± 0.03 (11)	0.31 ± 0.04 (8)	0.859	0.33 ± 0.02 (20)	0.29 ± 0.02 (14)	0.08
**NEFA (mmol/l)**	0.35 ± 0.03 (11)	0.34 ± 0.02 (8)	0.96	0.41 ± 0.03 (20)	0.34 ± 0.03 (14)	0.072
**Cholesterol (mmol/l)**	1.60 ± 0.08 (11)	1.94 ± 0.14 (8)	< 0.05	2.64 ± 0.21 (20)	2.93 ± 0.14 (14)	0.310
**Glucose (mmol/l)**	7.8 ± 0.4 (11)	7.6 ± 0.4 (8)	0.784	8.3 ± 0.5 (20)	7.7 ± 0.3 (14)	0.516
**Insulin (μg/l)**	0.21 ± 0.03 (11)	0.25 ± 0.02 (8)	0.383	0.46 ± 0.06 (17)	0.31 ±0.06 (14)	0.076
**HOMA-IR**	1.58 ± 0.28 (11)	2.01 ± 0.17 (8)	0.227	4.13 ± 0.67 (17)	2.67 ± 0.56 (14)	0.147
**HOMA-β (%)**	22.18 ± 3.71 (11)	31.54 ± 4.3 (8)	0.118	52.03 ± 6.70 (17)	34.27 ± 4.79 (14)	< 0.05
**QUICKI**	0.366 ± 0.010 (11)	0.345 ± 0.004 (8)	0.1	0.326 ± 0.010 (17)	0.344 ± 0.009 (14)	0.203

NEFA = non-esterified fatty acids, HOMA-IR = homeostatic model assessment of insulin resistance, HOMA-β homeostatic model assessment of beta-cell function, QUICKI = quantitative insulin sensitivity check index. Values expressed as mean ± SEM. Number of mice in each experiment indicated in the parenthesis. P-values by Student’s t-test or Mann-Whitney U-test.

### Serum cholesterol, triglycerides and fatty acids

Serum lipid profile of the VEH exposed OE-NPY^DβH^ and WT offspring at 7 months are presented in Table 1. When assessing the effect of metformin, OE-NPY^DβH^ female offspring had elevated total cholesterol compared to VEH exposed OE-NPY^DβH^ female offspring (Table 2). MET exposed OE-NPY^DβH^ male offspring had a tendency to declined circulating triglycerides (P = 0.08) and non-esterified fatty acids (NEFA, P = 0.07) in comparison to VEH exposed OE-NPY^DβH^ male offspring (Table 2).

### Gut microbiota

**Fig 4:**
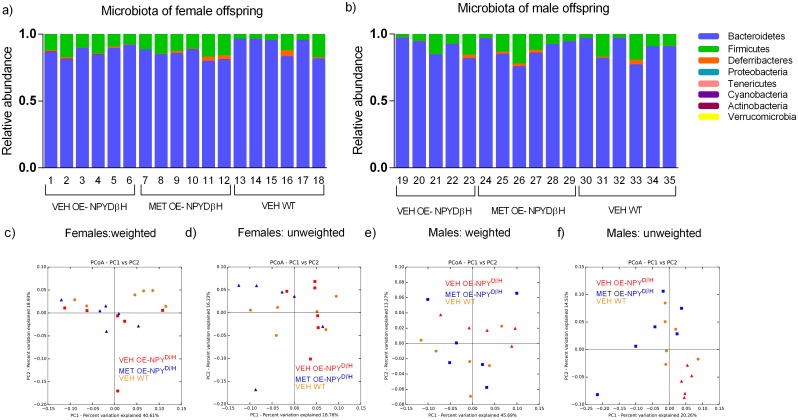
Composition of the gut microbiota. Occupation of microbiota by 8 most prominent phyla in the VEH and MET exposed OE-NPY^DβH^ and VEH exposed WT female and male offspring at 10–11 weeks (a,b). Weighted PCoA of the VEH OE-NPY^DβH^ vs. MET OE-NPY^DβH^ vs. VEH WT female offspring microbiota where principal coordinates PC1 explains 40.61% and PC2 18.93% of the total variance (c). Unweighted PCoA of the VEH OE-NPY^DβH^ vs. MET OE-NPY^DβH^ vs. VEH WT female offspring microbiota where PC1 explains 18.76% and PC2 16.23% of the total variance (d). Weighted PCoA of the VEH OE-NPY^DβH^ vs. MET OE-NPY^DβH^ vs. VEH WT male offspring microbiota where PC1 explains 45.69% and PC2 13.27% of the total variance (e). Unweighted PCoA of the VEH OE-NPY^DβH^ vs. MET OE-NPY^DβH^ vs. VEH WT male offspring microbiota where PC1 explains 20.26% and PC2 14.51% of the total variance (f). Red squares and triangles = VEH OE-NPY^DβH^ female (*n* = 6) and male (*n* = 5) offspring, respectively; blue triangles and squares = MET OE-NPY^DβH^ female (*n* = 6) and male (*n* = 6), offspring, respectively; orange circles = VEH WT female (*n* = 6) and male (*n* = 6) offspring. Plots produced by Qiime.

To determine the gut microbiota composition of the VEH and MET exposed OE-NPY^DβH^ and VEH exposed WT offspring, 16S rRNA sequencing was utilized. On the phylum level, the microbiota consisted mostly of *Bacteroidetes* and *Firmicutes* and to a lesser extent of *Deferribacteres*, *Proteobacteria*, *Tenericutes* and *Cyanobacteria*. The level of *Actinobacteria* and *Verrucomicrobia* were negligible (Fig 4a and 4b). Shannon index describing the diversity of the microbiota was not significantly affected by the genotype or by metformin exposure (data not shown). PCoA plots showed that VEH exposed OE-NPY^DβH^ and WT offspring differentiated from one another in both genders (Fig 4c–4f) whereas MET exposed OE-NPY^DβH^ offspring intermingled between VEH exposed OE-NPY^DβH^ and VEH exposed WT offspring (Fig 4c–4f). The independent effects of the genotype on the microbiota are presented in Table 3. The most prominent effect was observed on the abundance of *Proteobacteria* that was increased in the VEH exposed OE-NPY^DβH^ offspring in comparison to VEH exposed WT offspring (Mann-Whitney U-test).

**Table 3:** The gut microbiota comparison between VEH exposed OE-NPY^DβH^ and WT offspring at 10–11 weeks.

Gut microbiota	Female offspring			Male offspring		
	OE-NPY^DβH^	WT	P-value	OE-NPY^DβH^	WT	P-value
	MA	MA		MA	MA	
***Proteobacteria*** (phylum)	0.30%	0.08%	< 0.01	0.42%	0.18%	0.08
***Betaproteobacteria*** (class)	0.26%	0.07%	< 0.01	0.37%	0.13%	0.05
***Sutterella*** (genus)	0.26%	0.07%	< 0.01	0.37%	0.13%	0.05
***Bacteroidetes*** (phylum)						
***Bacteroidales; [Paraprevotellaceae]*** (family)	4.18%	9.78%	< 0.05	6.77%	11.85%	0.05
***Firmicutes*** (phylum)						
***Lachnospiraceae*** (family)	1.09%	0.35%	< 0.05	0.64%	0.75%	0.87
***Rikenellaceae*** (family)	3.40%	2.18%	0.09	2.01%	2.39%	0.52

MA = Mean abundance (%). P-values by Mann-Whitney U-test. *n* = 6 in the VEH exposed OE-NPY^DβH^ and WT female offspring and *n* = 5 and 6 in the VEH exposed OE-NPY^DβH^ and WT male offspring, respectively.

**Fig 5:**
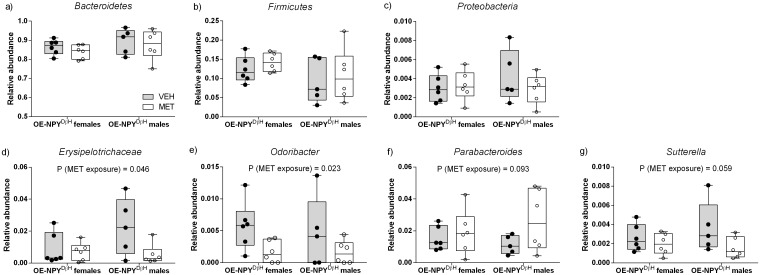
Relative abundancies of gut bacteria at different taxonomical levels at 10–11 weeks. Relative abundance of *Bacteroidetes* (a), *Firmicutes* (b), *Proteobacteria* (c), *Erysipelotrichaceae* (d), *Odoribacter* (e), *Parabacteroides* (f) and *Sutterella* (g) in the VEH OE-NPY^DβH^ (gray boxes) and MET OE-NPY^DβH^ (white boxes) female and male offspring. Relative abundance expressed as decimal number where 1 corresponds to 100%. Box plots showing the 25^th^, 75^th^ percentile and median and the whiskers extending from minimum to maximum together with all data points, *n* = 5–6. Significances by 2-way ANOVA, *P < 0.05.

When the effects of prenatal metformin exposure were analysed, the abundance of *Firmicutes*, *Bacteroidetes* or *Proteobacteria* (Fig 5a–5c) was not altered on the phylum level in the MET exposed OE-NPY^DβH^ offspring. However, one microbial family and three genera showed significant responses or a tendency to metformin exposure when genders were analysed together (2-way ANOVA): *Erysipelotrichaceae* (*Firmicutes;* P < 0.05), *Odoribacter* (*Bacteroidetes;* P < 0.05), *Parabacteroides* (*Bacteroidetes;* P = 0.093) and *Sutterella* (*Proteobacteria;* P = 0.059) (Fig 5d–5g).

**Fig 6:**
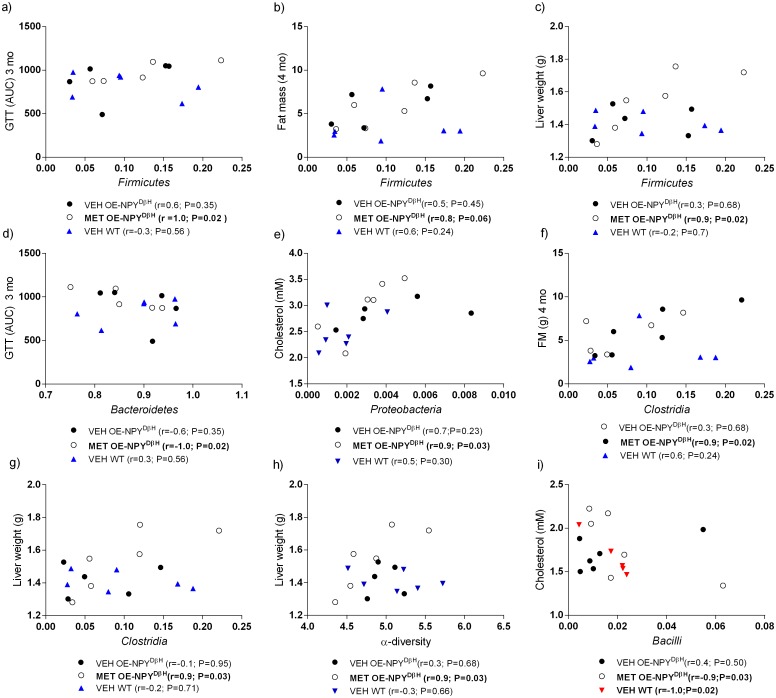
Correlation of gut microbiota composition to metabolic parameters. Correlation of the abundance of *Firmicutes* with GTT at 3 months (a), fat mass at 4 months (b), liver weight at 7 months (c), *Bacteroidetes* with GTT at 3 months (d), *Proteobacteria* with cholesterol at 7 months (e), *Clostridia* with fat mass at 4 months (f) and liver weight at 7 months (g) and α-diversity with liver weight at 7 months (h) in the VEH OE-NPY^DβH^ (*n* = 5), MET OE-NPY^DβH^ (*n* = 6) and VEH WT male (*n* = 6) offspring. Correlation of the abundance of *Bacilli* with serum cholesterol at 7 months (i) in the VEH OE-NPY^DβH^ (*n* = 6), MET OE-NPY^DβH^ (*n* = 6), and VEH WT (*n* = 6) female offspring. Black circles = VEH OE-NPY^DβH^, open circles = MET OE-NPY^DβH^, red and blue triangles = WT female and male offspring, respectively. P-values and r by Spearman correlation.

MET exposed OE-NPY^DβH^ male offspring were also found to have correlations between different taxonomic level microbiota and metabolic parameters. Abundance of *Firmicutes* correlated with GTT at 3 months (Spearman r = 1.0, P < 0.05, Fig 6a), with fat mass at 4 months (Spearman r = 0.8, P = 0.06, Fig 6b) and with liver weight at 7 months (Spearman r = 0.9, P < 0.05, Fig 6c). Moreover, the abundance of *Bacteroidetes* correlated negatively with GTT at 3 months (Spearman r = -1.0, P < 0.05, Fig 6d), *Proteobacteria* with serum cholesterol at 7 months (Spearman r = 0.9, P < 0.05, Fig 6e), *Clostridia* with fat mass at 4 months (Spearman r = 0.9, P < 0.05, Fig 6f), and liver weight at 7 months (Spearman r = 0.9, P < 0.05, Fig 6g) and α-diversity with liver weight at 7 months (Spearman r = 0.9, P < 0.05, Fig 6h). Of interest, the correlations between *Erysipelotrichi* and fat mass at 4 months and liver weight at 7 months and furthermore, between *Sutterella* and liver weight at 7 months were found only in the WT offspring (data not shown). In comparison to the male offspring, female offspring did not have similar correlations. Interestingly in the OE-NPY^DβH^ female offspring, the abundance of *Bacilli* correlated with cholesterol levels in the MET exposed offspring (Spearman r = -0.9, P < 0.05, Fig 6i) and in the WT offspring (Spearman r = -1.0, P < 0.05, Fig 6i).

The 16S rRNA sequencing data was also used for functional analyses utilising previously published method, PiCRUSt [48]. While only few KEGG pathways were found to be below non-adjusted P < 0.05 (Mann-Whitney U-test), none reached significance when P-values were corrected (FDR). The results of the functional analyses are presented in S1–S4 Tables.

## Discussion

The study shows that prenatal metformin exposure effectively modifies the metabolic phenotype of the OE-NPY^DβH^ mice. This mouse model was used due to its well characterized metabolic impairments [28,29], and its linkage to stress-induced obesity [35], a condition affecting humans in modern lifestyle. In the female offspring, the metabolic effects induced by metformin exposure were manifested by impairment in glucose tolerance, increase in fat mass and serum cholesterol whereas in the male offspring, there was a strong tendency of decline in fat mass accumulation, serum triglyceride and insulin levels. It was also shown that metformin exposure slightly modulated the composition of the gut microbiota at the age period when the metabolic impairments begin to manifest in the OE-NPY^DβH^ mice.

Our previous results on mice have shown that when metformin is given throughout the gestation, the maternal metabolic status attributes to the way metformin exposure translates to the phenotype of the offspring [12,14]. In the current study, a clear sexual dimorphism was observed in the metformin exposed offspring. The metabolic response to metformin exposure in the OE-NPY^DβH^ male offspring was similar in comparison with the metformin exposed offspring in the high fat diet model [14]. On the contrary, the metabolic phenotype of the metformin exposed OE-NPY^DβH^ female offspring was impaired, similar to that of the metformin exposed offspring in the normal diet model [12]. The cause of the sexual dimorphism remains vague but the exacerbated metabolic response of the female offspring is supported by a study where OE-NPY^DβH^ female mice manifest exacerbated obesity and metabolic impairments in comparison to WT when placed on a western diet at an early age [49]. Therein, the resistance to metabolic disturbances normally associated with female gender (i.e. estrogen) [50] is nullified both by early exposure to western-diet [49] and prenatal metformin exposure in the OE-NPY^DβH^ female offspring as shown here. Based on the current and previous studies, it is evident that different study designs lead to different phenotypes when investigating the long-term effects of metformin. Moreover, this is not different from the spectrum of results obtained in humans [9–11,51].

Impairment of glucose homeostasis was detected in the metformin exposed female offspring already at 3 months on regular diet. Glucose tolerance did not significantly correlate to body weight or fat mass (at 4 months) suggesting that the effects were independent of fat mass accumulation. The difference in the glucose tolerance was attenuated during the western diet as the response in glucose tolerance to the diet was more pronounced in vehicle exposed in comparison to metformin exposed OE-NPY^DβH^ female offspring. Glucose tolerance was not significantly affected in the male offspring. However, a tendency to decreased insulin levels and a significant difference in the HOMA-β index implicated improved insulin sensitivity in the metformin exposed offspring at 7 months. Furthermore, glucose tolerance and fasting insulin correlated with fat mass during WD implicating that the metformin-induced attenuation of fat mass accumulation in the male offspring is associated with glucose homeostasis.

Metformin exposed OE-NPY^DβH^ female offspring had significantly higher cholesterol levels compared to the vehicle exposed OE-NPY^DβH^ female offspring. Relevantly, we have previously shown that metformin exposed female offspring of the normal gestation have elevated cholesterol levels [12]. Moreover, in that study, prenatal metformin exposure was shown to associate with increased expression of hepatic *Insig 1* which has a potent function in cholesterol metabolism *via* sterol-mediated inhibition of cholesterol synthesis [52]. In humans, young metformin exposed children have on the contrary shown to have a tendency to lower LDL-cholesterol levels [53] and there are data that metformin treatment lowers LDL cholesterol in humans independently of the effects on glucose level [54]. The potential activation of cholesterol pathways during prenatal period and its impact on the metabolic programming of cholesterol metabolism justifies further investigation.

The gut microbiota of the vehicle exposed OE-NPY^DβH^ offspring in comparison to the vehicle exposed WT offspring had not been investigated earlier. NPY overexpression *per se* induced microbial alterations shown by moderately divergent clustering of the fecal samples in PCoA from vehicle exposed OE-NPY^DβH^ and WT offspring. Particularly, the abundance of *Proteobacteria* was increased in the OE-NPY^DβH^ offspring compared to the WT offspring. *Proteobacteria* has been reported to increase during high fat diet intervention in mice [55] and in obese and diabetic *db*/*db* mice [56]. Moreover, an increase in *Proteobacteria* and its subsequent decrease by dietary intervention have been reported in obese human subjects [57]. Therein, OE-NPY^DβH^ mice presented a suitable model to investigate the effects of prenatal metformin exposure on the gut microbiota. Indeed, the role of gut microbiota regarding the effects of prenatal metformin exposure has remained unanswered although the bacteria transmitted during gestation and the delivery to the fetus may have a significant impact on the development of child’s microbiota and later health [58–60]. In our study, metformin exposure had a greater effect on the microbiota of the male offspring in comparison to the female offspring according the PCoA. Undoubtedly, sex-hormone dependent modulation of the microbiota is present [61] and may also affect the magnitude of the alterations. The gut microbiota of the metformin exposed offspring in comparison to the vehicle exposed did not show any differences in the abundance of *Firmicutes* or *Bacteroidetes*, the two dominating phyla in the gut microbiota. This is intriguing as it has been previously shown that obesity is associated with higher abundance of *Firmicutes* and lower of *Bacteroidetes* in humans and in mice [62,63]. However, despite the lack of changes on the phylum level, the abundances of the gut microbes on the phyla level (including *Firmicutes and Bacteroidetes*) correlated with metabolic parameters in the male offspring exposed prenatally to metformin implicating that the level of the microbes has a predictive value in the metformin exposed male offspring. Regarding the changes on other taxonomic levels, the abundance of *Erysipelotrichaceae* was declined in metformin exposed OE-NPY^DβH^ offspring, particularly in the male gender. High abundance of class *Erysipelotrichi* has been connected to progression of non-alcoholic fatty liver disease (NAFLD) in choline-deficient diet in humans [64] and thus the decline in *Erysipelotrichaceae* may present an important treatment effect of metformin. Furthermore, our findings on *Odoribacter* and *Parabacteroides* indicate direct beneficial effects of metformin on the metabolism as *Odoribacter* has been found to be increased and *Parabacteroides* to be decreased in *db*/*db* mice [56], i.e. the opposite what was found here on metformin exposure. Moreover, increased prevalence of *Sutterella* has been detected in antibiotic-induced dysbiosis [65] but also upon prebiotic treatment in mice [66] making the decreased abundance of *Sutterella* somewhat conflicting. Altogether it appears that the gut microbial effects of prenatal metformin exposure were beneficial but at the same time the metabolism of the female offspring was impaired for a yet undefined cause.

There are some limitations in our study. Regarding the gut microbiota, the number of samples in the gut microbiota analyses was small and the intragroup variation relatively high. More samples per group might have revealed significant differences that now remained on the border of tendencies. Moreover, molecular analyses are needed to further pinpoint the mechanism although it has been implicated that early microbiota composition can be used as a prediction of future weight gain [67]. Regarding the metabolic phenotype, the minor decrease in the food intake and maternal body weight gain of the metformin administered OE-NPY^DβH^ dams during gestation may provide a possible contributing factor to the changes observed in the offspring. Hypophagic effect of metformin may be mediated through increased GLP-1 levels [18,19,68] and decreased hypothalamic NPY and Agouti-related peptide expression [69] although these responses were not studied here. Moreover, 3-day mating protocol might have introduced variation in the time of the fertilization and thus parturition between the groups.

In summary, we observed gender specific metabolic responses to prenatal metformin exposure in OE-NPY^DβH^ mice. Against our original hypothesis, prenatal metformin exposure had an additive effect to transgenic overexpression of NPY in the metabolic phenotype of the female offspring. On the contrary, metformin exposed male offspring had improvements in the metabolism that were in line with our previous data of the high fat diet model [14]. The current study suggests that prenatal metformin exposure has yet uncharacterized effects on the metabolism of the offspring that may also partly involve gut microbiota.

## Supporting Information

S1 Table Predicted pathways by PICRUSt in the VEH exposed OE-NPY^DβH^ vs. VEH exposed WT female offspring. (PDF)

S2 Table Predicted pathways by PICRUSt in the VEH vs. MET exposed OE-NPY^DβH^ female offspring. (PDF)

S3 Table Predicted pathways by PICRUSt in the VEH exposed OE-NPY^DβH^ vs. VEH exposed WT male offspring. (PDF)

S4 Table Predicted pathways by PICRUSt in the VEH vs. MET exposed OE-NPY^DβH^ male offspring. (PDF)
