# Supplementary material for: Neuropeptide Y Overexpressing Female and Male Mice Show Divergent Metabolic but Not Gut Microbial Responses to Prenatal Metformin Exposure
Source: PLoS One. 2016 Sep 28;11(9):e0163805. doi: 10.1371/journal.pone.0163805 (PMC5040270; doi:10.1371/journal.pone.0163805)
Supplement: S1 Table — (PDF) [file pone.0163805.s001.pdf]

S1 Table. Predicted pathways by PICRUSt in the VEH exposed OE-NPY$^{D\beta H}$ vs. VEH exposed WT female offspring.

	P-value (unadjusted)	FDR (adjusted P-value)
**Metabolism; Xenobiotics Biodegradation and Metabolism; 1,1,1-Trichloro-2,2-bis(4-chlorophenyl)ethane (DDT) degradation**	0.004	0.633
**Metabolism; Energy Metabolism; Nitrogen metabolism**	0.015	0.633
**Metabolism; Metabolism of Terpenoids and Polyketides; Geraniol degradation**	0.026	0.633
**Genetic Information Processing; Folding, Sorting and Degradation; Protein processing in endoplasmic reticulum**	0.041	0.633
**Cellular Processes; Cell Growth and Death; Apoptosis**	0.065	0.633
**Unclassified; Metabolism; Glycan biosynthesis and metabolism**	0.065	0.633
**Metabolism; Amino Acid Metabolism; Valine, leucine and isoleucine degradation**	0.065	0.633
**Metabolism; Xenobiotics Biodegradation and Metabolism; Bisphenol degradation**	0.093	0.633
**Metabolism; Energy Metabolism; Carbon fixation pathways in prokaryotes**	0.093	0.633
**Metabolism; Lipid Metabolism; Glycerolipid metabolism**	0.093	0.633
**Human Diseases; Cancers; Pathways in cancer**	0.093	0.633
**Human Diseases; Neurodegenerative Diseases; Prion diseases**	0.093	0.633

n(VEH OE-NPY$^{D\beta H}$) = 6, n(VEH WT) = 6. Unadjusted P-value by Mann-Whitney U-test.
