# Supplementary material for: Neuropeptide Y Overexpressing Female and Male Mice Show Divergent Metabolic but Not Gut Microbial Responses to Prenatal Metformin Exposure
Source: PLoS One. 2016 Sep 28;11(9):e0163805. doi: 10.1371/journal.pone.0163805 (PMC5040270; doi:10.1371/journal.pone.0163805)
Supplement: S2 Table — (PDF) [file pone.0163805.s002.pdf]

S2 Table. Predicted pathways by PICRUSt in the VEH exposed vs. MET exposed OE-NPY$^{D\beta H}$ female offspring.

	P-value (unadjusted)	FDR (adjusted P-value)
**Human Diseases; Cancers; Renal cell carcinoma**	0.041	0.985
**Metabolism; Biosynthesis of Other Secondary Metabolites; Caffeine metabolism**	0.061	0.985
**Human Diseases; Infectious Diseases; Chagas disease (American trypanosomiasis)**	0.061	0.985
**Organismal Systems; Environmental Adaptation; Circadian rhythm - plant**	0.061	0.985
**Metabolism; Lipid Metabolism; Fatty acid elongation in mitochondria**	0.061	0.985
**Metabolism; Lipid Metabolism; Steroid biosynthesis**	0.061	0.985
**Human Diseases; Immune System Diseases; Systemic lupus erythematosus**	0.061	0.985
**Organismal Systems; Circulatory System; Cardiac muscle contraction**	0.093	0.985
**Metabolism; Xenobiotics Biodegradation and Metabolism; Polycyclic aromatic hydrocarbon degradation**	0.093	0.985
**Metabolism; Biosynthesis of Other Secondary Metabolites; Stilbenoid, diarylheptanoid and gingerol biosynthesis**	0.093	0.985

n(VEH OE-NPY$^{D\beta H}$) = 6, *n*(MET OE-NPY$^{D\beta H}$) = 6. Unadjusted P-value by Mann-Whitney U-test.
