# Supplementary material for: Neuropeptide Y Overexpressing Female and Male Mice Show Divergent Metabolic but Not Gut Microbial Responses to Prenatal Metformin Exposure
Source: PLoS One. 2016 Sep 28;11(9):e0163805. doi: 10.1371/journal.pone.0163805 (PMC5040270; doi:10.1371/journal.pone.0163805)
Supplement: S3 Table — (PDF) [file pone.0163805.s003.pdf]

S3 Table. Predicted pathways by PICRUSt in the VEH exposed OE-NPY$^{D\beta H}$ vs. VEH exposed WT male offspring.

	P-value (unadjusted)	FDR (adjusted P-value)
Metabolism; Biosynthesis of Other Secondary Metabolites; Flavone and flavonol biosynthesis	0.017	0.947
Metabolism; Lipid Metabolism; Steroid hormone biosynthesis	0.030	0.947
Metabolism; Metabolism of Terpenoids and Polyketides; Biosynthesis of siderophore group nonribosomal peptides	0.052	0.947
Metabolism; Xenobiotics Biodegradation and Metabolism; Bisphenol degradation	0.052	0.947
Genetic Information Processing; Folding, Sorting and Degradation; Ubiquitin system	0.052	0.947
Unclassified; Metabolism; Carbohydrate metabolism	0.082	0.947
Metabolism; Lipid Metabolism; Glycerophospholipid metabolism	0.082	0.947
Metabolism; Metabolism of Terpenoids and Polyketides; Limonene and pinene degradation	0.082	0.947
Unclassified; Metabolism; Nucleotide metabolism	0.082	0.947
Unclassified; Genetic Information Processing; Transcription related proteins	0.082	0.947
Unclassified; Genetic Information Processing; Translation proteins	0.082	0.947

n(VEH OE-NPY$^{D\beta H}$) = 5, n(VEH WT) = 6. Unadjusted P-value by Mann-Whitney U-test.
