# Supplementary material for: Neuropeptide Y Overexpressing Female and Male Mice Show Divergent Metabolic but Not Gut Microbial Responses to Prenatal Metformin Exposure
Source: PLoS One. 2016 Sep 28;11(9):e0163805. doi: 10.1371/journal.pone.0163805 (PMC5040270; doi:10.1371/journal.pone.0163805)
Supplement: S4 Table — (PDF) [file pone.0163805.s004.pdf]

S4 Table. Predicted pathways by PICRUSt in the VEH exposed vs. MET exposed OE-NPY$^{D\beta H}$ male offspring.

	P-value (unadjusted)	FDR (adjusted P-value)
**Unclassified; Metabolism; Carbohydrate metabolism**	0.052	0.764
**Unclassified; Metabolism; Glycan biosynthesis and metabolism**	0.052	0.764
**Unclassified; Metabolism; Nucleotide metabolism**	0.052	0.764
**Metabolism; Carbohydrate Metabolism; Pentose and glucuronate interconversions**	0.052	0.764
**Unclassified; Genetic Information Processing; Translation proteins**	0.052	0.764
**Human Diseases; Infectious Diseases; African trypanosomiasis**	0.055	0.764
**Metabolism; Metabolism of Terpenoids and Polyketides; Biosynthesis of siderophore group nonribosomal peptides**	0.082	0.764
**Metabolism; Xenobiotics Biodegradation and Metabolism; Bisphenol degradation**	0.082	0.764
**Metabolism; Biosynthesis of Other Secondary Metabolites; Flavone and flavonol biosynthesis**	0.082	0.764
**Metabolism; Carbohydrate Metabolism; Pentose phosphate pathway**	0.082	0.764
**Genetic Information Processing; Translation; Ribosome Biogenesis**	0.082	0.764
**Metabolism; Lipid Metabolism; Steroid hormone biosynthesis**	0.082	0.764
**Unclassified; Genetic Information Processing; Transcription related proteins**	0.082	0.764
**Metabolism; Amino Acid Metabolism; Valine, leucine and isoleucine biosynthesis**	0.082	0.764

n(VEH OE-NPY$^{D\beta H}$) = 5, *n*(MET OE-NPY$^{D\beta H}$) = 6. Unadjusted P-value by Mann-Whitney U-test.
